# Assessment of the Risk of Insulin Resistance in Workers Classified as Metabolically Healthy Obese

**DOI:** 10.3390/nu17081345

**Published:** 2025-04-14

**Authors:** Miguel García Samuelsson, Pedro Juan Tárraga López, Ángel Arturo López-González, Hernán Paublini, Emilio Martínez-Almoyna Rifá, José Ignacio Ramírez-Manent

**Affiliations:** 1 ADEMA-Health Group, University Institute of Health Sciences (IUNICS), 07120 Palma de Mallorca, Balearic Islands, Spain; miguelsamuelsson@gmail.com (M.G.S.); h.paublini@eua.edu.es (H.P.); emilio@mompra.com (E.M.-A.R.); jignacioramirez@telefonica.net (J.I.R.-M.); 2 Faculty of Dentistry, ADEMA-UIB University School, 07009 Palma de Mallorca, Balearic Islands, Spain; 3 Faculty of Medicine, University of Castilla-La Mancha, 02071 Albacete, Spain; pjtarraga@sescam.jccm.es; 4 Balearic Islands Health Research Institute Foundation (IDISBA), 07004 Palma de Mallorca, Balearic Islands, Spain; 5 Balearic Islands Health Service, 07003 Palma de Mallorca, Balearic Islands, Spain; 6 Faculty of Medicine, University of the Balearic Islands, 07122 Palma de Mallorca, Balearic Islands, Spain

**Keywords:** metabolically healthy obese, insulin resistance, Mediterranean diet, physical activity, sociodemographic variables, tobacco consumption

## Abstract

**Introduction and Objectives**: Obesity constitutes a significant public health concern and is frequently linked to metabolic dysfunctions, particularly insulin resistance (IR). Nevertheless, a subset of obese individuals, referred to as metabolically healthy obese (MHO), do not exhibit overt metabolic abnormalities. The present study aims to assess the risk of developing IR among MHO workers and to explore the determinants contributing to this risk. **Methods**: This cross-sectional investigation utilized data from a cohort of 68,884 obese workers across multiple occupational sectors in Spain. The classification of participants as MHO was based on the number of metabolic syndrome components, in accordance with the criteria established by the National Cholesterol Education Program Adult Treatment Panel III (NCEP-ATPIII). Anthropometric, clinical, and biochemical parameters—including body mass index (BMI), waist circumference, lipid profile, glycemic levels, and blood pressure—were systematically assessed. The likelihood of developing IR was estimated through various validated risk assessment models. **Results**: The analysis indicates that, despite having a relatively favorable metabolic profile, individuals classified as MHO also show signs of metabolic deterioration, such as an increased risk of insulin resistance. Key risk factors such as physical inactivity, low adherence to the Mediterranean diet, and socioeconomic disparities were identified as significant contributors to the transition from the MHO phenotype to a metabolically unhealthy state. Logistic regression analyses corroborated that insufficient physical activity and suboptimal dietary habits were strongly associated with an elevated risk of IR. **Conclusions**: The findings underscore the dynamic and potentially transient nature of the MHO phenotype, emphasizing the necessity of proactive monitoring and early preventive strategies. Encouraging physical activity, promoting adherence to a nutritionally balanced diet, and implementing workplace health initiatives emerge as critical measures to attenuate the risk of IR and metabolic deterioration in MHO individuals. Future longitudinal studies are warranted to enhance risk stratification and to formulate tailored preventive interventions.

## 1. Introduction

Obesity represents one of the major public health challenges of the 21st century [1], frequently associated with various metabolic comorbidities, among which insulin resistance (IR) stands out [2]. However, a subgroup of obese individuals, referred to as “metabolically healthy obese” (MHO), appears not to exhibit the typical metabolic dis-turbances associated with excess adiposity [3]. This apparent paradox has garnered in-creasing interest within the scientific community, particularly concerning the risk of de-veloping IR in this population [4,5].

MHO individuals are defined as those with a body mass index (BMI) greater than 30 kg/m^2^ who, despite being obese, do not exhibit insulin resistance or other associated metabolic risk factors such as hypertension or hyperglycemia [6]. The most distinctive characteristic of these individuals is their higher insulin sensitivity compared to metabolically unhealthy obese (NMHO) individuals [7]. Additionally, they tend to have a more favorable lipid profile [8], lower levels of inflammatory markers [9], and a fat distribution that favors subcutaneous rather than visceral storage. This adipose tissue distribution may play a protective role against the development of metabolic disorders [10]. Epidemiological studies estimate that this phenotype accounts for approximately 10% to 30% of the obese population, depending on the diagnostic criteria employed and the demographic characteristics of the analyzed cohorts [11].

Insulin resistance is a condition in which muscle, fat, and liver cells do not respond adequately to insulin, hindering glucose uptake and leading to elevated blood sugar levels [12]. This dysfunction is a key factor in the pathogenesis of type 2 diabetes mellitus [13] and is associated with an increased risk of cardiovascular disease [14]. Several factors contribute to the development of IR, including visceral fat accumulation [15], chronic low-grade inflammation [16], and alterations in adipokine secretion [17]. Unlike subcutaneous fat, visceral fat exhibits higher lipolytic activity and releases free fatty acids into the portal system, negatively affecting liver function and promoting IR [18]. Additionally, visceral adipose tissue secretes proinflammatory cytokines that interfere with insulin signaling [19].

Although individuals with metabolically healthy obesity (MHO) exhibit a more favorable metabolic profile compared to those with non-metabolically healthy obesity (NMHO), evidence suggests that this condition may be transient [20]. Various studies have indicated that MHO is not an entirely benign phenotype, as many individuals classified within this category may transition to a metabolically unhealthy state over time. The dynamic nature of MHO, which differentiates between individuals with a stable profile and those transitioning to NMHO, could explain the discrepancies observed in the literature. Additionally, research has shown that individuals with MHO have a higher risk of developing metabolic abnormalities over time, such as insulin resistance, dyslipidemia, and hypertension, increasing their vulnerability to cardiovascular and metabolic diseases [21,22]. Therefore, longitudinal follow-up is essential to identify individuals at greater risk of progression to a less favorable state and to design appropriate intervention strategies.

Epigenetic studies using biomarkers indicate that a significant proportion of MHO individuals develop IR and other metabolic complications over time [23]. Findings from the CORDIOPREV study, with a 5-year follow-up, revealed that 71.8% of people with MHO moved into the NMHO category [24]. The transition from an MHO phenotype to a metabolically unhealthy state may be influenced by factors such as aging [25], additional weight gain [26], physical inactivity [27], and genetic predisposition [28]. Furthermore, the ability of subcutaneous adipose tissue to expand and store excess energy without inducing inflammation or fibrosis appears to be a crucial determinant in maintaining metabolic health in obese individuals [29].

The early identification of IR in MHO individuals is essential for implementing pre-ventive strategies to avoid progression toward NMHO states. The Homeostasis Model Assessment (HOMA) index is a widely used tool for estimating IR in clinical and epidemiological studies [30]. Research in pediatric populations has demonstrated that the HOMA index is useful in distinguishing between metabolically healthy and unhealthy obesity, suggesting its applicability across different age groups [31].

In addition to IR assessment, it is important to consider other clinical and biochemical markers, such as adiponectin levels [32], C-reactive protein [33], and lipid profiles [34], for a more comprehensive characterization of the metabolic status of MHO individuals. Adiponectin, in particular, has been associated with increased insulin sensitivity [35] and a lower cardiovascular risk [36], with higher levels observed in MHO individuals compared to their NMHO counterparts.

Recognizing the metabolic heterogeneity among obese individuals has significant implications for clinical practice. While MHO individuals have a lower immediate risk of metabolic complications, they should not be considered risk free. Regular monitoring of metabolic parameters and the promotion of healthy lifestyle habits, including a balanced diet and regular physical activity, are essential for maintaining metabolic health and preventing the onset of IR and other comorbidities.

Interventions aimed at improving adipose tissue function, such as strategies to en-hance the storage capacity of subcutaneous adipose tissue and reduce inflammation, may be beneficial in preventing the transition from an MHO phenotype to a NMHO state. Additionally, identifying genetic and molecular markers that predict susceptibility to developing IR in MHO individuals could facilitate the implementation of personalized interventions.

The objective of this study is to assess the level of IR risk in a cohort of workers classified as MHO.

## 2. Methods

### 2.1. Study Design and Participants

A cross-sectional descriptive analysis was conducted using data from occupational medical examinations performed on 68,884 obese Spanish workers (45,498 men and 23,386 women) across the primary, secondary, and tertiary sectors between January 2019 and June 2020.

Inclusion Criteria:

- Obesity, defined as a body mass index (BMI) ≥ 30 kg/m^2^.
- Age between 18 and 69 years.
- Employment at one of the participating companies.
- Voluntary participation in the study.

Exclusion Criteria:

- Individuals younger than 18 or older than 69 years.
- No employment contract with any participating company.
- Did not provide informed consent to participate in the study.
- Did not authorize the use of their data for epidemiological purposes.
- Missing variables necessary for calculations.
- Body mass index (BMI) ≤ 30 kg/m^2^.

**Figure 1:**
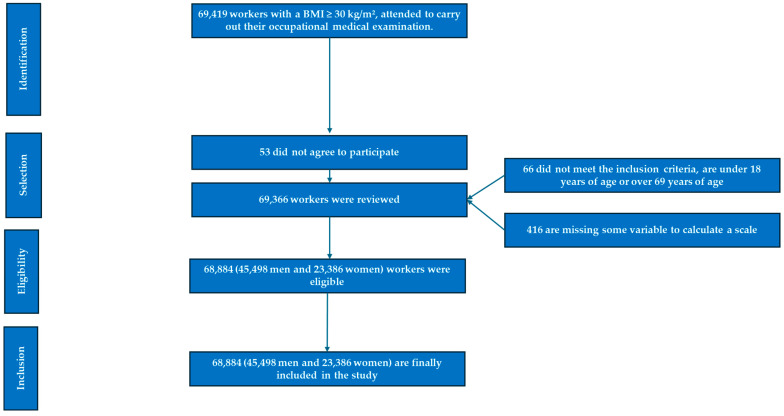
Flowchart of the participants in the study.

The participant flowchart is presented in Figure 1.

### 2.2. Variable Assessment

After standardizing measurement techniques, the study’s medical and nursing personnel conducted clinical, analytical, and anthropometric assessments, including waist circumference, weight, and height.

Body weight and height were measured using a SECA 700 scale (SECA, Chino, CA, USA) and a SECA 220 stadiometer (SECA, Chino, CA, USA). Participants were barefoot and wearing underwear, following ISAK guidelines for anthropometric assessments [37]. Waist circumference was assessed with a SECA measuring tape (SECA, Chino, CA, USA), ensuring proper positioning: participants stood upright, with feet together and abdomen relaxed. The tape was placed parallel to the floor at the level of the lowest floating rib [38].

Blood pressure was recorded using a calibrated OMRON M3 automatic sphygmo-manometer (OMRON, Osaka, Japan) while participants remained seated after a minimum rest period of 10 min. Three consecutive measurements were taken at 60 s intervals, and the final recorded value corresponded to the average of these readings.

### 2.3. Biochemical Analyses

Following a fasting period of at least 12 h, biochemical parameters were assessed. Concentrations of total cholesterol, glucose, and triglycerides were determined through automated enzymatic techniques. High-density lipoprotein cholesterol (HDL-c) levels were obtained using dextran sulfate-MgCl_2_ precipitation methods. Low-density lipoprotein cholesterol (LDL-c) was indirectly estimated using the Friedewald equation [39], expressed in mg/dL:

LDL-c = Total Cholesterol − HDL-c + Triglycerides/5

Obesity was defined as a body mass index (BMI) ≥ 30 kg/m^2^.

### 2.4. Definition of Metabolically Healthy Obesity (MHO)

To classify individuals as metabolically healthy obese (MHO), the criteria established by the National Cholesterol Education Program Adult Treatment Panel III (NCEP-ATPIII) for metabolic syndrome (MS) were applied [40]. These criteria included the presence of at least one of the following metabolic risk factors:

- Waist circumference: ≥88 cm in women and ≥102 cm in men.
- Triglyceride levels: ≥150 mg/dL or undergoing lipid-lowering therapy.
- HDL cholesterol levels: <50 mg/dL in women or <40 mg/dL in men.
- Fasting glucose levels: ≥100 mg/dL or receiving glucose-lowering treatment.
- Blood pressure status: Systolic blood pressure (SBP) ≥ 130 mmHg and/or diastolic blood pressure (DBP) ≥ 85 mmHg, or the use of antihypertensive therapy.

Participants were subsequently categorized into three groups based on the number of metabolic syndrome components present:

- Group A: No metabolic syndrome factors.
- Group B: One metabolic syndrome factor.
- Group C: Up to two metabolic syndrome factors.

### 2.5. Demographic and Socioeconomic Variables

- Sex was recorded as a binary variable (male or female).
- Age was determined by subtracting the date of birth from the date of the medical ex-amination.
- Educational attainment was categorized into three levels: Primary education, High school education, and University education.
- Socioeconomic status was classified according to the Spanish Society of Epidemiolo-gy criteria, based on the 2011 National Occupational Classification (CNO-11) [41], and categorized as follows:
  ○ Social Class I: Executives, university-educated professionals, athletes, and artists.
  ○ Social Class II: Intermediate professionals and skilled self-employed workers.
  ○ Social Class III: Low-skilled workers.

Lifestyle and Behavioral Variables

- Participants were classified as smokers if they had consumed any form of tobacco at least once per day in the past 30 days or had ceased smoking within the preceding 12 months.
- Adherence to the Mediterranean diet was evaluated using a 14-item questionnaire, with responses scored as 0 or 1 point per item. A total score ≥ 9 was indicative of high adherence [42].
- Physical activity levels were assessed using the International Physical Activity Questionnaire (IPAQ), a self reported instrument designed to quantify physical activity patterns over the previous 7 days [43].

### 2.6. Statistical Analysis

Descriptive statistics were employed to summarize categorical variables, expressed as frequencies and distributions, while the normality of continuous variables was assessed using the Kolmogorov–Smirnov test. Means and standard deviations were calculated for quantitative variables. Bivariate analyses included a Student’s t-test for mean comparisons and chi square tests for proportion analysis. To examine factors associated with metabolically healthy obesity (MHO), a multinomial logistic regression model was applied, with model fit assessed using the Hosmer–Lemeshow test. Stratified analyses were conducted to control for potential confounders, but no significant effects were detected. All statistical procedures were executed using SPSS (version 29.0), with statistical significance set at *p* < 0.05.

### 2.7. Ethical Considerations

This study was conducted in accordance with ethical research standards, including compliance with the 2013 Declaration of Helsinki. The anonymity and confidentiality of participants were strictly safeguarded throughout the study. Ethical approval was granted by the Balearic Islands Research Ethics Committee (CEI-IB) under reference number IB 4383/20, approved on 26 November 2020. All participant data were coded, ensuring that only the principal investigator had access to personally identifiable information. The majority of researchers involved in the study adhered to Organic Law 3/2018 (December 5) on Personal Data Protection and Digital Rights, guaranteeing that participants retained the right to access, rectify, erase, and object to the processing of their data at any time.

## 3. Results

Table 1 presents the characteristics of the study participants, highlighting that an-thropometric, clinical, and analytical variables show less favorable values in men. The predominant age group in this study falls between 30 and 49 years. Most workers belong to the lowest socioeconomic levels (social class III) and have only primary education. Physical activity levels and adherence to the Mediterranean diet are notably low. More than 31% of men and slightly more than one in four women are smokers.

In all cases, the differences observed between men and women are statistically sig-nificant (*p* < 0.001).

In Table 2, we present the mean values of the scales used to estimate the risk of de-veloping insulin resistance or prediabetes. Meanwhile, Table 3 provides the prevalence of elevated values for these same scales, comparing these values between the MHO) and NMHO groups. In both cases, the same trend is observed, namely, higher values in the NMHO group. The differences observed across all scales consistently show statistical significance (*p* < 0.001).

Table 4 presents the findings of the multinomial logistic regression analysis, demon-strating that all independent variables incorporated into the model—including sex, age, educational attainment, social class, smoking status, physical activity levels, adherence to the Mediterranean diet, and classification as either MHO or NMHO (based on the three established criteria)—exhibit significant associations with elevated scores on the insulin resistance and prediabetes risk scales. Among these factors, the most pronounced associa-tions, as indicated by odds ratios (ORs), are observed for physical activity, adherence to the Mediterranean diet, and metabolic classification as MHO or NMHO.

## 4. Discussion

The findings of this study provide relevant evidence on the association between the risk of insulin resistance (IR) and prediabetes in workers classified as MHO. Although the MHO phenotype has been considered a condition with a lower metabolic risk, the results support the idea that individuals with MHO are not exempt from developing metabolic disorders.

The cross-sectional design prevents the establishment of causal relationships, as data are collected at a single time point, making it difficult to distinguish between causality and correlation. Additionally, selection bias may arise if the sample does not adequately represent the population. However, in our case, we consider that the large sample size reduces this bias.

One of the most significant findings is that, despite presenting a more favorable metabolic profile compared to NMHO individuals, workers with MHO also show signs of metabolic deterioration, as evidenced by the values of IR risk scales. In particular, physical activity [44,45,46] and adherence to the Mediterranean diet [47,48,49] have been identified as key protective factors against IR, highlighting the importance of promoting healthy lifestyle habits in this population.

Adipose tissue distribution appears to play a crucial role in the transition from the MHO phenotype to a NMHO state [50]. Previous studies have demonstrated that preferential fat storage in subcutaneous adipose tissue, rather than visceral adipose tissue, is associated with a lower metabolic risk [51]. However, over time, the capacity of subcutaneous adipose tissue to store excess energy is exceeded, leading to increased lipotoxicity and systemic inflammation, both of which are key factors in the development of IR [52].

Another aspect to consider is the influence of sociodemographic and lifestyle factors on the metabolic evolution of MHO individuals. In this study, age, educational level, and social class were found to be associated with a higher risk of IR and prediabetes [53,54,55], indicating that social inequalities may play a significant role in the progression of MHO to a pathological state. These findings align with previous research suggesting that limited access to healthy foods [56], reduced opportunities for physical activity [57], and chronic stress [58] contribute to metabolic deterioration in vulnerable populations.

It would be of great interest to further explore the relationship between metabolic health and the organizational level of workers within each occupational category, considering classifications such as directors, managers, technicians, officers, and laborers. This analysis would help identify whether specific patterns exist in the distribution of healthy lifestyle habits according to occupational hierarchy and determine whether certain organizational levels present a higher metabolic risk.

Additionally, it would be relevant to describe the predominant employment profiles, differentiating between manual labor, sedentary work, and shift work. Each of these occupational types involves different physical demands and stress levels, which could influence the prevalence of insulin resistance and other metabolic disorders.

This approach would provide a better understanding of how workplace characteristics impact workers’ health, including factors such as available time for physical activity, access to healthy nutrition, and exposure to stressors. The interaction between occupational type, job-related physical demands, lifestyle habits, and access to health resources could offer valuable insights for designing workplace health promotion and prevention strategies.

Therefore, this analysis represents a key aspect to consider in future research on occupational health and the risk of developing insulin resistance.

From a clinical perspective, these results highlight the need for periodic monitoring of MHO individuals to identify early signs of transition toward IR and other metabolic complications such as prediabetes. The use of tools such as the Homeostatic Model Assessment (HOMA) index for evaluating insulin resistance [59], along with biomarkers such as adiponectin [60] and C-reactive protein [61], could allow for more precise stratification of metabolic risk in this population. Combining these markers with imaging techniques, such as magnetic resonance imaging (MRI) [62] or computed tomography (CT) [63], could provide a more detailed assessment of the metabolic status of MHO individuals. However, these data are not available in our study and should be considered in future research.

Physical activity plays a crucial role in preventing insulin resistance and the development of prediabetes, as evidenced by the findings from the multinomial logistic regression analysis. The results show significant associations between high scores on insulin resistance scales and the risk of prediabetes, with physical activity acting as a protective factor. This is reflected in the Odds Ratio values, which range from 3.88 to 6.78, indicating that individuals with lower physical activity levels have a significantly higher probability of developing insulin resistance compared to those who engage in regular exercise.

This protective effect can be explained by several physiological mechanisms. Physical activity enhances insulin sensitivity by increasing glucose uptake in skeletal muscles, thereby reducing blood glucose levels. Additionally, exercise promotes a reduction in visceral adiposity, which is closely linked to chronic inflammation and metabolic dysfunction. It also influences the regulation of adipokines and inflammatory cytokines, modulating metabolic responses and lowering the risk of developing insulin resistance [64].

The findings of this study reinforce the importance of promoting physical activity as a key strategy for preventing metabolic diseases. Including specific recommendations on exercise frequency, intensity, and type could help develop more effective interventions in at-risk populations [65]. Since physical activity is a modifiable factor, its promotion in workplaces, educational settings, and community programs could be an effective strategy to reduce the burden of type 2 diabetes and other related metabolic diseases. These findings highlight the need for further research in this area to optimize prevention strategies.

The Mediterranean diet is a fundamental pillar in preventing metabolic disorders, particularly insulin resistance. The results from the multinomial logistic regression analysis indicate that lack of adherence to this dietary pattern significantly increases the risk of developing insulin resistance, with Odds Ratio values ranging from 2.90 to 5.35. This suggests that individuals who do not follow the Mediterranean diet have nearly three to more than five times the likelihood of developing insulin resistance compared to those who adhere to it.

The metabolic benefits of the Mediterranean diet can be attributed to its composition, which is rich in monounsaturated fatty acids from olive oil, a high intake of fruits, vegetables, legumes, and nuts, and a moderate consumption of fish and dairy products. These foods contain bioactive compounds that reduce systemic inflammation, oxidative stress, and improve insulin sensitivity. Additionally, dietary fiber from fruits, vegetables, and whole grains modulates the glycemic response and supports the gut microbiome balance, contributing to better metabolic regulation [66].

This study reinforces the importance of promoting the Mediterranean diet as a key strategy in preventing insulin resistance and related metabolic disorders such as type 2 diabetes and metabolic syndrome. Nutritional education and public health policies that encourage adherence to this dietary pattern could be essential in reducing the incidence of these conditions. Furthermore, personalized interventions could be designed to improve Mediterranean diet adherence, particularly in populations with high-risk factors. These results underscore the need for continued research on the relationship between nutrition and metabolic health.

Smoking is established as a modifiable risk factor in the development of insulin resistance, as evidenced by the results of the multinomial logistic regression analysis. Our analysis shows an increased risk of insulin resistance ranging from 3% to 16% in smokers compared to non-smokers, highlighting the importance of addressing this habit in the prevention of metabolic disorders.

The pathophysiological mechanisms underlying this relationship include oxidative stress and chronic inflammation induced by toxic tobacco components, which impair endothelial function and disrupt insulin signaling [67]. Additionally, smoking is associated with an unfavorable distribution of body fat, promoting the accumulation of visceral fat—a key factor in insulin resistance [68].

Since smoking is a modifiable risk factor, its control should be a priority in public health strategies. Implementing smoking cessation programs and raising awareness of its metabolic effects could significantly reduce the risk of developing insulin resistance and associated metabolic diseases.

Furthermore, it is essential to design intervention strategies aimed at improving the metabolic health of MHO individuals. Interventions that promote physical activity maintenance, the adoption of a healthy diet, and stress reduction could play a crucial role in preventing the development of IR [69,70]. In this regard, implementing health promotion programs in the workplace could be an effective strategy for improving workers’ wellbeing and preventing the progression of MHO to a pathological state [71]. Strategies that include personalized nutritional counseling, tailored physical training, and stress management programs could be particularly beneficial for this population group.

The role of genetics and epigenetics in the evolution of MHO individuals is another factor that warrants special attention [72]. Recent studies have demonstrated that certain genetic variants may predispose individuals to transition to an NMHO state [28]. Additionally, epigenetic regulation induced by environmental factors such as diet and physical exercise can modulate the expression of genes related to inflammation [73], glucose metabolism, and insulin sensitivity [74]. Research in this field could open new avenues for the development of personalized therapies aimed at preventing the progression of IR in these individuals.

It is important to note that variability in the diagnostic criteria for the MHO phenotype poses a challenge for comparability across studies. Different studies have used varying thresholds to define insulin resistance and other metabolic risk factors, which may influence the estimated prevalence of this phenotype and the interpretations of its clinical impact [75,76]. Standardizing these criteria and incorporating longitudinal analyses would facilitate a better understanding of the evolution of MHO individuals and the factors contributing to their transition to a metabolically unhealthy state.

Another key aspect is the impact of public health policies on the prevention and management of the MHO phenotype. Government initiatives aimed at promoting healthy lifestyles, improving access to nutritious foods, and reducing work-related stress and bur-dens among workers could play a crucial role in reducing the incidence of IR in this pop-ulation [77,78]. Collaboration between the health, education, and labor sectors could yield more effective strategies for promoting long-term metabolic health.

Among the essential strategies to improve employee health, several concrete actions can be implemented in the workplace. First, adequate health education training for all workers is fundamental to promoting the adoption of healthy habits and preventing chronic diseases. The promotion of healthy lifestyles should be supported by awareness programs tailored to the needs of each occupational group.

Second, the implementation of workplace nutrition policies is key to improving employees’ dietary habits. This includes offering healthy meal options in workplace cafeterias and eliminating vending machines that promote the consumption of ultra-processed foods. Another essential measure is ensuring work schedules that allow employees sufficient time to eat properly, preventing rushed or unbalanced meals due to a lack of breaks. Additionally, the installation of fitness areas in the workplace would encourage regular physical activity, benefiting cardiovascular health and overall well-being.

Finally, companies can develop physical exercise and stress management programs, such as mindfulness, yoga, and relaxation techniques. These strategies would not only improve individual health and well-being but could also enhance productivity and company profitability by reducing absenteeism and improving employee performance.

### Strengths and Limitations

A key strength of this study is its large sample size, comprising nearly 69,000 obese workers, making it one of the most extensive investigations of MHO conducted globally. Another notable advantage is the wide range of analyzed variables, encompassing both sociodemographic and lifestyle-related factors, and their relationship with MHO. Furthermore, the use of validated questionnaires to assess physical activity levels and adherence to the Mediterranean diet enhances the study’s reliability, offering a cost-effective and practical approach for evaluation and longitudinal monitoring.

However, this study is subject to certain limitations. One of the primary constraints is its cross-sectional design, which precludes the ability to establish causal relationships. Additionally, the exclusion of unemployed individuals, retirees, and those under 18 or over 69 years of age limits the generalizability of the findings to the broader population. Nevertheless, the large sample size is expected to mitigate this limitation to some extent. Similarly, as the study was conducted exclusively within a Spanish population, the findings may not be directly applicable to other populations, necessitating caution in extrapolating the results.

Not stratifying by hierarchical level within each occupation limits the analysis of patterns according to job hierarchy and specific sectors. Understanding how the work environment, physical demands, and access to health resources influence lifestyle habits is essential for designing preventive strategies and guiding future research in occupational health.

The lack of biomarkers is another limitation of our work, as they would be very useful in allowing for more precise metabolic risk stratification in this population.

Another potential limitation stems from the use of self-administered questionnaires, which are inherently susceptible to recall bias and social desirability bias. Future research should consider integrating objective validation methods to enhance data accuracy. Additionally, certain confounding factors, such as comorbidities and pharmacological treatments, were not accounted for due to data unavailability, which may have influenced the outcomes.

## 5. Conclusions

The results of this study reinforce the evidence that the MHO phenotype should not be considered a stable or benign condition, but rather a transitory state that may evolve into metabolic dysfunction over time. Early identification of IR risk and the implementation of prevention strategies based on the promotion of healthy lifestyles are essential to minimize the complications associated with obesity in this population. Adopting a comprehensive approach that combines clinical assessments, advanced biomarkers, genetic and epigenetic studies, and personalized intervention strategies could be key to improving long-term metabolic outcomes in MHO individuals.

## Figures and Tables

**Table 1:** Characteristics of the participants.

	Men n = 45,498	Women n = 23,386	
	Mean (SD)	Mean (SD)	*p*-Value
Age (years)	42.9 (10.0)	42.0 (10.4)	<0.001
Height (cm)	173.2 (7.1)	160.0 (6.7)	<0.001
Weight (kg)	99.7 (12.4)	87.5 (12.3)	<0.001
Waist (cm)	96.7 (8.9)	83.3 (8.8)	<0.001
Hip (cm)	108.6 (7.9)	109.5 (9.3)	<0.001
Systolic BP (mmHg)	131.8 (16.2)	124.0 (15.9)	<0.001
Diastolic BP (mmHg)	81.0 (10.7)	76.9 (11.0)	<0.001
Total cholesterol (mg/dL)	204.1 (38.8)	200.3 (37.4)	<0.001
HDL-cholesterol (mg/dL)	48.3 (7.0)	51.2 (7.1)	<0.001
LDL-cholesterol (mg/dL)	124.5 (37.5)	127.1 (37.0)	<0.001
Triglycerides (mg/dL)	158.6 (108.4)	110.5 (55.8)	<0.001
Glucose (mg/dL)	92.3 (14.0)	89.0 (13.4)	<0.001
	%	%	*p*-Value
<30 years	10.0	13.5	<0.001
30–39 years	28.3	28.2	
40–49 years	34.5	32.3	
50–59 years	22.6	21.9	
60–69 years	4.6	4.3	
Elementary school	63.7	64.9	<0.001
High school	32.3	30.6	
University	4.0	4.5	
Social class I	4.6	4.2	<0.001
Social class II	15.7	21.4	
Social class III	79.7	74.4	
No physical activity	96.5	95.3	<0.001
Physical activity	3.5	4.7	
No Mediterranean diet	91.8	85.1	<0.001
Mediterranean diet	8.2	14.9	
Non-smokers	68.3	74.0	<0.001
Smokers	31.7	26.0	

BP blood pressure. HDL High density lipoprotein. LDL Low density lipoprotein. SD Standard deviation.

**Table 2:** Mean values of different insulin resistance risk scales in metabolically healthy and unhealthy obese to sex.

	n = 8764	n = 36,734		n = 24,264	n = 21,234		n = 34,660	n = 10,838	
	MHO (A)	NMHO (A)		MHO (B)	NMHO (B)		MHO (C)	NMHO (C)	
Men	Mean (SD)	Mean (SD)	*p*-Value	Mean (SD)	Mean (SD)	*p*-Value	Mean (SD)	Mean (SD)	*p*-Value
TyG index	8.3 (0.3)	8.9 (0.6)	<0.001	8.4 (0.4)	9.0 (0.6)	<0.001	8.5 (0.5)	9.1 (0.6)	<0.001
TyG-BMI	264.7 (19.6)	297.6 (38.2)	<0.001	270.9 (23.3)	303.5 (38.6)	<0.001	278.7 (27.1)	313.0 (40.0)	<0.001
TyG-waist	774.5 (52.8)	880.1(105.1)	<0.001	794.9 (65.5)	898.6(103.7)	<0.001	822.0 (78.8)	926.6(103.5)	<0.001
TyG-WtHR	4.5 (0.3)	5.1 (0.6)	<0.001	4.6 (0.4)	5.2 (0.6)	<0.001	4.7 (0.4)	5.3 (0.6)	<0.001
METS-IR	45.6 (3.4)	52.6 (7.0)	<0.001	47.0 (4.1)	53.8 (7.0)	<0.001	48.7 (4.8)	55.8 (7.2)	<0.001
SPISE-IR	2.1 (0.2)	2.5 (0.5)	<0.001	2.1 (0.3)	2.6 (0.5)	<0.001	2.2 (0.3)	2.7 (0.5)	<0.001
PRISQ	17.4 (6.8)	26.8 (8.4)	<0.001	20.4 (7.7)	28.1 (8.0)	<0.001	22.7 (8.0)	29.9 (7.6)	<0.001
Women	n = 6146	n = 17,240		n = 14,446	n = 8938		n = 19,976	n = 3410	
TyG index	8.1 (0.4)	8.5 (0.5)	<0.001	8.2 (0.4)	8.6 (0.5)	<0.001	8.3 (0.4)	8.7 (0.5)	<0.001
TyG-BMI	257.2 (15.5)	293.1 (40.6)	<0.001	267.3 (25.2)	300.0 (41.6)	<0.001	276.4 (31.3)	311.2 (43.3)	<0.001
TyG-waist	659.4 (47.0)	775.6 (97.2)	<0.001	702.6 (75.2)	793.8 (95.6)	<0.001	730.9 (84.3)	820.5 (95.5)	<0.001
TyG-WtHR	4.2 (0.3)	4.8 (0.6)	<0.001	4.4 (0.4)	4.9 (0.6)	<0.001	4.6 (0.5)	5.1 (0.6)	<0.001
METS-IR	43.2 (2.2)	50.4 (6.9)	<0.001	45.4 (4.4)	51.7 (7.0)	<0.001	47.3 (5.2)	53.6 (7.3)	<0.001
SPISE-IR	1.9 (0.2)	2.4 (0.5)	<0.001	2.1 (0.3)	2.4 (0.5)	<0.001	2.2 (0.4)	2.6 (0.5)	<0.001
PRISQ	15.1 (6.5)	23.6 (8.3)	<0.001	17.5 (7.0)	25.3 (8.0)	<0.001	19.9 (7.6)	27.6 (7.7)	<0.001

TyG Triglyceride glucose index. BMI Body mass index. WtHR Waist to height ratio. METS-IR Metabolic score for insulin resistance. SPISE-IR Single-point insulin sensitivity estimator-insulin resistance. MHO Metabolically healthy obese. NMHO Non-Metabolically healthy obese. (A) 0 factors of metabolic syndrome. (B) <2 factors of metabolic syndrome (C) <3 factors of metabolic syndrome.

**Table 3:** Prevalence of high values of different insulin resistance risk scales in metabolically healthy and unhealthy obese to sex.

	n = 8764	n = 36,734		n = 24,264	n = 21,234		n = 34,660	n = 10,838	
	MHO (A)	NMHO (A)		MHO (B)	NMHO (B)		MHO (C)	NMHO (C)	
Men	%	%	*p*-Value	%	%	*p*-Value	%	%	*p*-Value
TyG index high	3.1	52.2	<0.001	11.7	61.0	<0.001	24.4	74.6	<0.001
METS-IR high	9.4	58.9	<0.001	18.3	67.8	<0.001	32.1	80.3	<0.001
SPISE-IR high	21.5	71.3	<0.001	33.8	79.2	<0.001	48.1	88.9	<0.001
PRISQ high	5.3	51.8	<0.001	19.9	58.1	<0.001	30.7	67.6	<0.001
Women	n = 6146	n = 17,240		n = 14,446	n = 8938		n = 19,976	n = 3410	
TyG index high	5.0	31.2	<0.001	7.9	38.1	<0.001	13.4	52.8	<0.001
METS-IR high	0.3	42.2	<0.001	11.0	50.7	<0.001	22.9	63.1	<0.001
SPISE-IR high	5.9	55.8	<0.001	22.2	64.5	<0.001	35.8	76.3	<0.001
PRISQ high	2.7	25.7	<0.001	5.1	31.8	<0.001	10.3	44.4	<0.001

TyG Triglyceride glucose index. METS-IR Metabolic score for insulin resistance. SPISE-IR Single-point insulin sensitivity estimator for insulin resistance. PRISQ Prediabetes Risk Score in Qatar. MHO Metabolically healthy obese. NMHO Non-Metabolically healthy obese. (A) 0 factors of metabolic syndrome. (B) <2 factors of metabolic syndrome (C) <3 factors of metabolic syndrome.

**Table 4:** Multinomial logistic regression.

	TyG Index High	SPISE-IR High	METS-IR High	PRISQ High
	OR (95% CI)	OR (95% CI)	OR (95% CI)	OR (95% CI)
Female	1	1	1	1
Male	2.25 (2.14–2.35)	1.22 (1.18–1.26)	1.33 (1.26–1.40)	1.36 (1.28–1.45)
<30 years	1	1	1	1
30–39 years	1.06 (1.03–1.09)	1.09 (1.06–1.13)	1.16 (1.09–1.23)	1.10 (1.06–1.14)
40–49 years	1.11 (1.07–1.15)	1.29 (1.20–1.38)	1.35 (1.28–1.42)	1.21 (1.16–1.26)
50–59 years	1.24 (1.12–1.37)	1.59 (1.43–1.75)	1.68 (1.50–1.86)	1.85 (1.61–2.19)
60–69 years	1.61 (1.43–1.81)	2.08 (1.80–2.36)	1.99 (1.80–2.19)	2.11 (1.88–2.34)
University	1	1	1	1
High school	1.09 (1.06–1.12)	1.12 (1.06–1.18)	1.08 (1.04–1.12)	1.07 (1.04–1.10)
Elementary school	1.20 (1.13–1.27)	1.28 (1.16–1.40)	1.25 (1.18–1.32)	1.23 (1.15–1.31)
Social class I	1	1	1	1
Social class II	1.05 (1.02–1.08)	1.21 (1.15–1.27)	1.06 (1.03–1.10)	1.16 (1.12–1.20)
Social class III	1.29 (1.20–1.38)	1.53 (1.37–1.70)	1.33 (1.24–1.42)	1.41 (1.31–1.51)
Physical activity	1	1	1	1
No physical activity	6.34 (5.98–6.70)	5.29 (4.96–5.62)	6.78 (6.30–7.26)	3.88 (3.59–4.18)
Mediterranean diet	1	1	1	1
No Mediterranean diet	5.35 (4.99–5.71)	3.40 (3.05–3.75)	5.10 (4.70–5.51)	2.90 (2.49–3.31)
Non-smokers	1	1	1	1
Smokers	1.06 (1.03–1.10)	1.16 (1.11–1.20)	1.14 (1.09–1.18)	1.03 (1.00–1.07)
MHO (A)	1	1	1	1
NMHO (A)	4.67 (4.25–5.12)	2.12 (2.02–2.21)	2.05 (1.94–2.17)	2.21 (2.07–2.36)
MHO (B)	1	1	1	1
NMHO (B)	5.52 (5.27–5.79)	2.98 (2.86–3.11)	2.62 (2.51–2.74)	2.36 (2.18–2.54)
MHO (C)	1	1	1	1
NMHO (C)	8.79 (8.24–9.37)	8.81 (8.10–9.58)	4.25 (4.03–4.47)	7.60 (6.59–8.77)

TyG Triglyceride glucose index. METS-IR Metabolic score for insulin resistance. SPISE-IR Single-point insulin sensitivity estimator for insulin resistance. PRISQ Prediabetes Risk Score in Qatar. MHO Metabolically healthy obese. NMHO Non-Metabolically healthy obese. (A) 0 factors of metabolic syndrome. (B) <2 factors of metabolic syndrome (C) <3 factors of metabolic syndrome. OR Odds ratio.

## Data Availability

Data are not available due to ethical or privacy restrictions. This study’s data are stored in a database that complies with all security measures at the ADEMA-Escuela Universitaria. The Data Protection Delegate is Ángel Arturo López González.
